# Excellence Through Collaboration - an Audit of the Joint Perinatal Mental Health Clinic in Milton Keynes University Hospital

**DOI:** 10.1192/bjo.2026.11713

**Published:** 2026-06-30

**Authors:** Justyna Gromala, Joyce Elliot

**Affiliations:** Milton Keynes University Hospital, Milton Keynes, United Kingdom

## Abstract

**Aims:**

Every fifth pregnant person experiences mental health problems during pregnancy and the puerperium. The Joint Perinatal Mental Health Clinic (JPMHC) at Milton Keynes University Hospital was established to offer multidisciplinary team (MDT) support to patients with a complex mental health background during this time. Here, patients are supported by a team, including a psychiatrist, an obstetrician and a specialised midwife. Plans are made for pregnancy, delivery and follow-up. We aimed to audit the outcomes of this clinic between July 2023 – July 2024.

**Methods:**

Data were extracted from electronic patient records, for all patients booked into the JPMHC in July 2023 – July 2024. Data were analysed and figures were generated using Microsoft Excel.

**Results:**

35 patients were given an appointment with 83% attending all their appointments. Patients booked into clinic were 29.5 years old on average (19-39). 17% patients were from ethnic minority backgrounds, 74% were from a white background and 9% from other backgrounds.

Of the 32 patients that attended at least one appointment, 94% were seen by at least 3 MDT members. 74% of these patients had a perinatal mental health plan recorded. 28% were seen with at least one relative present.

82% of all patients had two or more psychiatric diagnoses recorded. The most common mental health backgrounds were affective disorders (31 diagnoses), anxiety disorders (29 diagnoses) and history of trauma or adverse life experiences (31 diagnoses). 69% of patients were on at least one psychotropic medication. 26% were on 2 or more psychotropic medications. The most used medications were SSRIs (16 prescribed), and antipsychotics (11 prescribed).

**Conclusion:**

This audit highlights the value of MDT input to address the needs of pregnant patients with complex mental health backgrounds. Being seen in a joint clinic allowed for clear communication with patients, their relatives and between relevant healthcare teams. This allowed for detailed perinatal mental health plans to be made. Close collaboration in the MDT promotes safety, for example when considering teratogenicity risks or adverse effects on neonates with the use of psychotropic medication. MDT members provide expert guidance on patients in their care, which is essential in the context of psychotropic polypharmacy and a multitude of psychiatric diagnoses.

The JPMHC helps pregnant people look after their physical and mental health as well as their growing child’s wellbeing in a vulnerable time in life. Going forward, we aim to design a standardised template and create a clinic model for other sites to implement.

